# Dyspnea in Post-Acute COVID-19: A Multi-Parametric Cardiopulmonary Evaluation

**DOI:** 10.3390/jcm12144658

**Published:** 2023-07-13

**Authors:** Antonella Cecchetto, Gabriella Guarnieri, Gianpaolo Torreggiani, Andrea Vianello, Giulia Baroni, Chiara Palermo, Leonardo Bertagna De Marchi, Giulia Lorenzoni, Patrizia Bartolotta, Emanuele Bertaglia, Filippo Donato, Patrizia Aruta, Sabino Iliceto, Donato Mele

**Affiliations:** 1 Department of Cardiac Thoracic Vascular Sciences and Public Health, University of Padua, 35128 Padua, Italy; torreggianigianpaolo@gmail.com (G.T.); baroni.giu@gmail.com (G.B.); chiara.palermo@unipd.it (C.P.); giulia.lorenzoni@unipd.it (G.L.); patrizia.bartolotta@ubep.unipd.it (P.B.); bertagliaferro@gmail.com (E.B.); filippo.donato@aopd.veneto.it (F.D.); patrizia.aruta@aopd.veneto.it (P.A.); sabino.iliceto@unipd.it (S.I.); donato.mele@unipd.it (D.M.); 2 Respiratory Pathophysiology Division, University of Padua, 35128 Padua, Italy; gabriella.guarnieri@aopd.veneto.it (G.G.); andrea.vianello@aopd.veneto.it (A.V.); leonardo.bertagnademarchi@studenti.unipd.it (L.B.D.M.)

**Keywords:** dyspnea, post-acute COVID-19, RVGLS, RVGLS/sPAP, respiratory muscle strength

## Abstract

Post-acute COVID-19 is characterized by the persistence of dyspnea, but the pathophysiology is unclear. We evaluated the prevalence of dyspnea during follow-up and factors at admission and follow-up associated with dyspnea persistence. After five months from discharge, 225 consecutive patients hospitalized for moderate to severe COVID-19 pneumonia were assessed clinically and by laboratory tests, echocardiography, six-minute walking test (6MWT), and pulmonary function tests. Fifty-one patients reported persistent dyspnea. C-reactive protein (*p* = 0.025, OR 1.01 (95% CI 1.00–1.02)) at admission, longer duration of hospitalization (*p* = 0.005, OR 1.05 (95% CI 1.01–1.10)) and higher body mass index (*p* = 0.001, OR 1.15 (95% CI 1.06–1.28)) were independent predictors of dyspnea. Absolute drop in SpO_2_ at 6MWT (*p* = 0.001, OR 1.37 (95% CI 1.13–1.69)), right ventricular (RV) global longitudinal strain (*p* = 0.016, OR 1.12 (95% CI 1.02–1.25)) and RV global longitudinal strain/systolic pulmonary artery pressure ratio (*p* = 0.034, OR 0.14 (95% CI 0.02–0.86)) were independently associated with post-acute COVID-19 dyspnea. In conclusion, dyspnea is present in many patients during follow-up after hospitalization for COVID-19 pneumonia. While higher body mass index, C-reactive protein at admission, and duration of hospitalization are predictors of persistent dyspnea, desaturation at 6MWT, and echocardiographic RV dysfunction are associated with this symptom during the follow-up period.

## 1. Introduction

The clinical course of COVID-19 in its acute phase is now delineated in sufficient detail; instead, less is known about its late phase [1]. The World Health Organization defines the post-acute sequelae of SARS-CoV-2 infection (PASC) as a condition that arises 12 weeks after infection, persists for at least eight weeks, and cannot be explained by alternative diagnoses [2].

Dyspnea is a common symptom of PASC with high incidence and a significant impact on quality of life [3,4]. The underlying mechanisms of dyspnea in this contest are still poorly understood and are probably multifactorial. Potential mechanisms include cardiac and pulmonary dysfunction, whereas pulmonary fibrosis and vascular changes after recovery have been described on chest-computed tomography (CT) [5,6]. Moreover, earlier reports showed an increased prevalence of high cardiac troponin levels, a biomarker of myocardial injury, which was associated with impaired left ventricular (LV) and right ventricular (RV) function, leading to higher morbidity and mortality rates, including possible long-term consequences [7].

Previous studies show conflicting results about pulmonary involvement. Dyspnea can persist despite improvements in cardiopulmonary function; in fact, it was demonstrated that at a three-month follow-up, the pulmonary function test parameters and chest CT abnormalities improved, independent of improvements in six-minute walk distance or dyspnea [8,9]. Another study reported the persistence of dyspnea, impaired six-minute walk distance, and reduced health-related quality of life, but without accompanying pulmonary function abnormalities [10]. Some studies showed no difference between healthy controls and patients with post-COVID dyspnea [11], and others found exercise intolerance with evidence of circulatory and breathing pattern abnormalities [12,13]. At least, it is not excluded that symptoms may be attributed to pre-existing conditions [14].

In this study, we decided to evaluate patients affected by moderate to severe COVID-19 pneumonia to establish the prevalence of dyspnea at follow-up and to identify clinical, laboratory, and instrumental factors at admission and follow-up associated with the persistence of dyspnea, to better clarify the underlying mechanisms. We hypothesized that patients with more severe pneumonia were more symptomatic at follow-up due to pulmonary compromise. Instead, no cardiac sequelae were associated with dyspnea. These findings would help clinicians to better understand the heterogeneous causes of persistent dyspnea after COVID-19 pneumonia in order to focus on the treatment.

## 2. Materials and Methods

### 2.1. Study Protocol

This is a single-center observational cohort study. The study was approved by the Ethics Committee on Human Research of Padua University (protocol code n. 20009). Because of the retrospective observational nature of the study, written consent was not required.

All patients had an established diagnosis of COVID-19 (positive polymerase chain reaction test of the nasopharyngeal swab or bronchoalveolar lavage fluid) and survived the acute event. Consecutive individuals recovering from COVID-19 pneumonia were evaluated in a dedicated outpatient clinic.

Inclusion criteria were: (1) COVID-19 pneumonia diagnosis as described above; (2) hospital discharge from the Respiratory Pathophysiology Unit of the Padua University Hospital between 18 February 2020 and 10 November 2021; (3) moderate to critical acute disease according to the National Institutes of Health definitions. Mild disease: mild clinical symptoms, no signs of pneumonia on imaging. Moderate disease: fever and respiratory symptoms, etc., with pneumonia signs on imaging. Severe disease: patients with any of the following conditions: respiratory distress with respiratory rate > 30 breaths/min; peripheral capillary oxygen saturation (SpO_2_) < 93% at rest; a ratio of arterial oxygen partial pressure (PAO_2_) to fractional inspired oxygen (FiO_2_) or PAO_2_/FiO_2_ < 300 mmHg. Critical disease: patients with respiratory failure requiring mechanical ventilation; shock; or other organ failure requiring admission to the Intensive Care Unit (ICU).

Exclusion criteria were: age < 18 years, poor-quality echocardiographic images, presence of dyspnea before admission, and anemia on blood test.

All patients underwent comprehensive medical assessment with a detailed medical history, physical examination, and blood tests (including full blood count, renal and liver function tests, troponin, B-type natriuretic peptide (BNP), C-reactive protein (CPR), and D-dimer). In particular, clinical examination assessed the persistence of self-reported dyspnea using the modified Medical Research Council (mMRC) score [15]. The scale ranges from 0 (no dyspnea) to 4 (severe dyspnea). Dyspnea was defined as an mMRC ≥ 1. We also performed a comprehensive cardiopulmonary evaluation that included complete rest transthoracic echocardiography, functional lung test, and six-minute walking test (6-MWT). We used a standardized data-collection tool to gather information from the patient’s electronic hospital records on demographics, body mass index (BMI), comorbidities, medication history, smoking history, severity of illness, maximum respiratory support requirements, length of hospitalization, and biomarkers relative to the acute phase of the disease (troponin, BNP, CPR, and D-dimer).

### 2.2. Transthoracic Echocardiography

The echocardiogram was performed on the same day as the clinical and pulmonary evaluation. Echocardiographic image acquisition was performed using a Vivid E9 echo scanner (GE Vingmed Ultrasound, Horten, Norway) by one experienced researcher. Data sets were digitally stored and exported on a dedicated workstation equipped with the EchoPac BT 13 software (GE Vingmed Ultrasound, Horten, Norway). A comprehensive assessment of biventricular and atrial size, LV diastolic function, LV and RV systolic function was performed using two-dimensional (2D) and three-dimensional (3D) conventional echocardiography and 2D speckle tracking echocardiography (2D STE) for longitudinal strain (LS) measurement, in accordance with the guidelines of the European Association of Cardiovascular Imaging (EACVI) and the American Society of Echocardiography (ASE) [16].

The following measures were obtained: the LV end-systolic volume, end-diastolic volume, and ejection fraction (EF); the ratio of early transmitral peak flow velocity (E wave) to late transmitral peak flow velocity (A wave) and the ratio of early transmitral peak E wave to early diastolic medial septal and lateral peak tissue Doppler velocity (e’); the left atrial volume; the right atrial volume and area; the RV end-diastolic area, end-systolic area, fractional area change, end-diastolic volume, end-systolic volume, and EF; the tricuspid annular plane systolic excursion; the systolic pulmonary artery pressure (sPAP); the pulmonary artery acceleration time; and the pulmonary vascular resistance. In addition, the probability of pulmonary hypertension was estimated in accordance with international guidelines [17]. Regarding LS analysis, LV global longitudinal strain (LVGLS), RV-free wall strain (RVFWS), RV global longitudinal strain (RVGLS), and its afterload correction (RVGLS/sPAP) were measured.

### 2.3. Pulmonary Functional Test

Pulmonary functional tests and lung diffusing capacity for carbon monoxide (DLCO) measurement were performed using calibrated equipment (MasterLab Pro; Erich Jaeger GmbH; Höchberg, Germany), according to the European Respiratory Society (ERS) and American Thoracic Society (ATS) recommendations [18,19]. The predicted normal values of Quanjer [20] and equations of Cotes for DLCO [21] were used. Among the various measures provided by the spirometry, vital capacity (VC), total lung capacity (TLC), DLCO, carbon monoxide transfer coefficient (KCO), and Tiffeneau index were included in the analysis. To assess respiratory muscles function, maximal inspiratory pressure (MIP) and expiratory mouth pressure (MEP) were measured [22]. For each patient, measures were also expressed as a percentage of the theoretical value.

### 2.4. Six-Minute Walking Test

The 6MWT was performed according to recommended guidelines [23], with baseline and after exercise, SpO_2_ measurement obtained by pulse oximetry on index fingers. Walking capacity was considered abnormal when below the cut-off value for a similar cohort of healthy patients (484 mt) [24]. Desaturation was defined as a drop of ≥4% or a reduction of <90% in SpO_2_.

### 2.5. Statistical Analysis

Data were reported as the median and interquartile range (IQR, I–III quartile) for continuous variables and as absolute values and percentages for categorical variables. The relationships among different measures were assessed using Spearman’s correlation. Univariable and multivariable logistic regression analyses were used to estimate the effect of different variables of the acute phase and follow-up on the persistence of dyspnea evaluated at follow-up. Multivariable model selection was done according to the Bayesian Information Criterion (BIC), starting from a set of candidate predictors. Such predictors were identified according to clinical judgment and literature review to be factors potentially associated with the outcome of interest. The BIC is a well-established methodology for model selection within clinical data [25]. The results were reported as odds ratios (ORs) with 95% confidence intervals (CIs). Statistical significance was taken as *p* < 0.05. Statistical analysis was performed using the R software version 4.1.0 222.

## 3. Results

### 3.1. Baseline Characteristics

From 18 February 2020 to 10 November 2021, during the first wave of the pandemic, 229 consecutive patients were initially enrolled. Three patients were excluded because of the poor quality of the echocardiographic images, and one patient died before the follow-up visit. Therefore, 225 patients were available for follow-up. All patients were Caucasians. The median time of assessment was 154 days (IQR 113–225) from hospitalization. Patients were mainly overweight adults without a history of cardiovascular disease but with at least one cardiovascular risk factor. During hospitalization, 188 (84%) received O_2_-therapy, and 56 (26%) patients underwent mechanical ventilation. All patients manifested signs of infection; 36 (16%) and 144 (64%) presented, respectively, troponin and BNP increased above the reference values. Patients’ characteristics are shown in Table 1, according to the presence of dyspnea at follow-up, and recently detailed in a previous publication [26].

### 3.2. Clinical Evaluation at Follow-Up

One hundred and fifteen patients (51%) reported persistent dyspnea five months after hospitalization. Of them, 43 (19%) reported an mMRC score = 1; 38 (17%) an mMRC score = 2; 22 (10%) an mMRC score = 3; and 12 (5%) an mMRC score = 4.

### 3.3. Cardiopulmonary Evaluation at Follow-Up

At the follow-up visit, 10% of patients presented LV dilatation, 9% LVEF reduction, 12% LV diastolic dysfunction, 28% RV dilatation, and 8% RVEF reduction. Thirty-six percent of patients had LVGLS reduction (>−18%), and 32% had RVGLS reduction (>−20%). Only 7% of the cohort demonstrated an intermediate and high probability of pulmonary hypertension. The values of transthoracic echocardiography measures at the follow-up visit are reported in Table 2.

Regarding the pulmonary functional test, patients were characterized by normal pulmonary volumes without signs of a lung-restrictive pattern. A trend toward reduction in lung diffusion capacity (55% of patients) and in respiratory muscle strength both during inhalation (28% of patients) and exhalation (64%) was observed. At the 6MWT, a reduction in the total walking distance occurred in 41% of patients and desaturation in 32% of patients (4% with SpO_2_ < 90% and 28% with a drop ≥4%). Among patients with a reduction of walking distance, 30% presented an absolute drop in SpO_2_ and 1% a final SpO_2_ < 90%. Only 4% of patients with final distance walked within normal limits presented an absolute drop in SpO_2_. The pulmonary functional test and 6MWT measures obtained at the follow-up evaluation are detailed in Table 3.

### 3.4. Acute Predictors of Persistent Dyspnea at Follow-Up

Clinical and laboratory characteristics of patients at hospitalization were tested for the association with persisting dyspnea during follow-up (Table 4).

In the multivariable analysis, higher BMI and longer hospitalization period significantly predicted the persistence of dyspnea during follow-up. Persistence of dyspnea during follow-up was also significantly and independently predicted by CRP.

### 3.5. Association between Persistent Dyspnea and Cardiopulmonary Measures at Follow-Up

In the univariable logistic regression analysis, persistent dyspnea was significantly associated with an alteration of respiratory parameters and especially with a decrease in lung diffusion capacity. Moreover, the presence of dyspnea was significantly associated with a reduction of respiratory muscle strength in the expiratory compartment. Distance and final saturation during the 6MWT were also significantly associated with the symptom. In the multivariable analysis, only the absolute drop in SpO_2_ at 6MWT, RVGLS, and RVGLS/sPAP was significantly associated with post-acute COVID-19 dyspnea. Results of the univariable and multivariable analysis for post-acute COVID-19 dyspnea are reported in Table 5.

## 4. Discussion

In this study, we evaluated a middle-aged cohort of patients affected by moderate to severe COVID-19 pneumonia, mostly with cardiovascular risk factors but without a history of cardiovascular disease. The main findings of this study are: (I) approximately half of the patients reported dyspnea during the first five months after hospitalization; (II) the severity of COVID-19 during the acute phase and being overweight predicted the persistence of dyspnea in the context of PASC; (III) the only echocardiographic measures associated with dyspnea during the follow-up period were RVGLS and RVGLS/sPAP.

Dyspnea is a peculiar symptom of the syndrome called PASC or “Long-COVID”. In our sample, 51% of patients experienced dyspnea during the first five months after hospitalization for COVID-19. In our study, dyspnea prevalence was slightly higher than previously observed (6,27). Carfì et al. [6] reported dyspnea in 43.4% of patients 60 days after the onset of the first COVID-19 symptom. Zheng et al. [27] described post-COVID breathlessness in 26% and 41% of patients, depending on the method used to evaluate it. These discrepancies could be attributable to the fact that our cohort included only hospitalized patients, characterized by moderate to critically ill related to COVID-19 infection, and to the different follow-up duration.

In our patients, a more severe COVID-19 during the acute phase (higher CRP and longer hospitalization) was predictive of dyspnea during the follow-up period, as observed in previous studies. Arnold et al. [28], for example, reported ongoing symptoms in 59%, 75%, and 89% of patients, respectively, with mild, moderate, and severe COVID-19, defined on the basis of the type of ventilation used. Conversely, we did not find a significant association between cardiovascular comorbidity and persistence of symptoms, differently from other studies [29,30,31], except for the association with higher BMI values [32]. These differences could be attributed to the heterogeneity of the patients considered in literature studies, characterized by different age, disease severity, type of symptoms, and individual comorbidities.

Regarding PASC, there is no clear consensus on whether dyspnea can be considered secondary to cardiopulmonary dysfunction or a self-reported symptom. In fact, in the literature studies, a minority of symptomatic patients had abnormal functional tests. Arnold et al. [28] described persistent symptoms (breathlessness and fatigue) in 74% of the patients 8–12 weeks post-admission; however, clinically significant abnormalities in chest radiographs, exercise tests, blood tests, and spirometry were less frequent (35%), especially in patients not requiring supplementary O_2_ during their acute infection (7%).

Mood abnormalities may also play a causative role in post-COVID dyspnea, in addition to cardiorespiratory abnormalities [33]. We tried to understand if dyspnea was objectively identifiable using functional tests and if it was associated with pulmonary or cardiac impairment. In our patients, desaturation at 6MWT was associated with dyspnea. In the literature investigations, the difference in walking distance was associated with significant subjective dyspnea [10]. These results define 6MWT as a reliable test to objectify dyspnea.

We confirmed a relationship between the persistence of dyspnea and an impairment of pulmonary function. In fact, the reduction of lung diffusion capacity and respiratory muscle strength was related to dyspnea. These results partially disagree with those of Lam et al. [10], who reported a PASC phenotype characterized by persistent dyspnea, and impaired 6MWT distance but without pulmonary function abnormalities. At the same time, the correlation between dyspnea and respiratory parameters alteration is consistent with part of the current literature studies [8,34]. Lerum et al. [8] confirmed a significant reduction of DLCO in 24% of patients three months after discharge, without differences between ICU and non-ICU participants. Robey et al. [34] confirmed an abnormal DLCO 4 months after COVID-19 infection, highest in the ICU cohort (64% ICU vs. 38% non-ICU patients).

Pathological changes in the diaphragm of COVID-19 patients post-mortem have been reported [35], and survivors from severe COVID-19 were observed to have impaired diaphragm contractility and diaphragmatic atrophy [36]. Furthermore, several studies have found decreased inspiratory muscle strength [37,38,39]. The correlation between dyspnea and expiratory muscle weakness that we described may be attributed to the occurrence of interstitial lung disease after COVID-19 or to physical deconditioning [40]. In fact, deconditioning was identified as a factor causing dyspnea in studies that evaluated persistent symptoms after COVID-19 using cardiopulmonary exercise testing [41]. COVID-19 infection is thought to cause muscle breakdown due to systemic inflammation, the so-called cytokine storm. Despite the limited literature studies that have examined MIP and MEP in patients with COVID-19, expiratory muscle weakness in dyspneic patients suggests the importance of target rehabilitation.

According to our findings, the rest echocardiographic measures were not associated with dyspnea, except for RVGLS and RVGLS/sPAP. The hearts of our patients presented normal LV and RV volumes, systolic and diastolic function, and a low probability of pulmonary hypertension in both patients with and without dyspnea. Beaudry et al. did not identify structural or functional cardiac changes in dyspneic patients; however, they did not evaluate subclinical myocardial injury and did not show respiratory alterations. This may be due to the fact that less severe non-hospitalized, young, non-obese, and comorbidity-free patients were enrolled [11]. The correlation between RVGLS impairment and the presence of dyspnea could be the consequence of more severe pulmonary disease as the result of increased stress inflicted on the RV in acute COVID-19. Ozer et al. previously demonstrated the relationship between the severity of pneumonia and subclinical impairment of RV function in hospitalized patients [42]. Tryfou et al. found impairment of RVGLS in patients hospitalized for COVID-19 pneumonia, while non-hospitalized patients had a normal RVGLS value [43]. RVGLS can be a predictive measure of severe disease, as suggested by a previous study, which confirmed that RVGLS was related to survival in patients with COVID-19 pneumonia, with a cut-off value of 20% [44].

Recent studies show that RV to pulmonary circulation coupling can be estimated non-invasively by echocardiography, using parameters of RV systolic function indexed to the sPAP, like TAPSE/sPAP and RVGLS/sPAP. The concept of RV to pulmonary circulation coupling refers to the relationship between RV contractility and RV afterload [45]. The RV, in contrast to the LV, is more susceptible to the increased afterload related to pulmonary diseases [46,47]. In this context, Polito et al. demonstrated that poor RV-arterial coupling, in terms of TAPSE/sPAP, may help to identify COVID-19 patients at higher risk of mortality during hospitalization [48]. Our study analyzed for the first time the role of indexing RVGLS to sPAP in the setting of patients recovered from COVID-19 pneumonia, demonstrating the association between persistent dyspnea with this measure. This suggests that interventions focusing on dyspnea management may be appropriate for the phenotype of post-acute COVID-19 patients. Only patients with a severe form of COVID-19-related pneumonia and persistence of respiratory function impairment could benefit from an instrumental evaluation of RV function with advanced transthoracic echocardiography techniques. The presence of RVGLS and RVGLS/sPAP abnormalities indicates the need for further cardiac and pulmonary evaluation and/or follow-up.

Some limitations of this study should be highlighted. Our study did not include a multi-parametric evaluation in the acute phase of hospitalization due to the restrictions posed by the COVID-19 infection. Instrumental examinations were not available prior to hospitalization. We included only hospitalized patients who successfully recovered from COVID-19, disregarding patients with higher severity who died during hospitalization. Cardiopulmonary stress testing was not performed to better understand the mechanisms involved in dyspnea. It might be useful to validate the results of the study by comparison with a population of COVID-19 patients with mild pulmonary impairment. However, at present, hospitalized COVID-19 patients have decreased considerably. Lastly, our longitudinal sample evaluation could be further improved by extending the follow-up.

## 5. Conclusions

We described a picture that falls under the PASC condition, documenting the persistence of dyspnea during the follow-up period in a significant number of hospitalized patients with COVID-19. Dyspnea was measurable by 6MWT and was associated with a respiratory function impairment, including a reduction of expiratory muscle strength. The only echocardiographic measures associated with dyspnea were RVGLS and RVGLS/sPAP.

## Figures and Tables

**Table 1:** Demographic and clinical characteristics at hospitalization according to the presence of dyspnea at follow-up.

Variables	Total Patients (*n* = 225)	Dyspnea Absent at FU (*n* = 110)	Dyspnea Present at FU (*n* = 115)	*p* Value
Age (y) (median IQR, (range))	65 (55, 73)	65 (54, 73)	65 (55, 72)	NS
Gender (n(%)) F	88 (39%)	36 (33%)	52 (45%)	NS
M	137 (61%)	74 (67%)	63 (55%)	
Smoke (n (%)) no	125 (56%)	64 (59%)	61 (53%)	NS
user	11 (5%)	8 (7.3%)	3 (2.6%)	
former smoker	88 (39%)	37 (34%)	51 (44%)	
Body mass index (kg/m^2^) (median IQR (range))	28.1 (24, 31.4)	28.1 (25.1, 31.2)	28.4 (24.5, 31.8)	NS
Previous cardiovascular disease (n (%))				NS
Absent	213 (95%)	106 ((96%)	107 (93%)	
Present	12 (5.3%)	4 (3.6%)	8 (7.0%)	
Type of cardiovascular disease (n (%))		-	-	
Ischemic	8 (3.5%)			
Valvular	3 (1.3%)			
Hypertrophic cardiomyopathy	1 (0.4%)			
Duration of hospitalization (days) (median IQR (range))	18 (12, 28)	17 (12, 25)	20 (13, 35)	NS
Respiratory ventilation (Type; (n (%))				NS
Absent	36 (16%)	12 (11%)	24 (21%)	
NC-SM	25 (11%)	12 (11%)	13 (11%)	
RM	6 (3%)	3 (2.8)	3 (2.6%)	
HFNC	69 (31%)	42 (40%)	27 (24%)	
NIV	28 (13%)	13 (12%)	15 (13%)	
MV	56 (26%)	24 (23%)	32 (28%)	
Troponin I (ng/L) (median IQR (range)) n.v. < 34 ng/L	9 (5, 26)	8 (4, 17)	12 (6, 65)	0.035
CRP (mg/l) (median IQR (range)) n.v. < 5 mg/L	120 (81, 180)	110 (78, 142)	135 (86, 218)	0.024
D-dimer (ng/mL) (median IQR (range)) n.v. < 500 ng/mL	662 (295, 1927)	564 (290, 1166)	767 (324, 2620)	NS
BNP (pg/mL) (median IQR (range)) n.v. < 125 pg/mL	70 (26, 120)	40 (16, 102)	80 (48, 210)	0.001
Cardiovascular risk factors (n (%))				NS
Absent	74 (33%)	36 (33%)	38 (33%)	
Present	151 (67%)	74 (67%)	77 (67%)	
Cardiovascular risk factors type (n (%))		-	-	
Hypertension	101 (45%)			
Dyslipidemia	33 (14%)			
Diabetes Mellitus	34 (15%)			
Obesity	58 (26%)			
CKD	7 (3%)			

B-type natriuretic peptide (BNP); chronic kidney disease (CKD); C-reactive protein (CRP); female (F); follow-up (FU); high-flow nasal cannula (HFNC); interquartile range (IQR); male (M); mechanical ventilation (MV); nasal cannula (NC); non-invasive ventilation (NIV); non-rebreather mask (RM); normal value (n.v.); not significant (NS); number (n); simple face mask (SM); years (y).

**Table 2:** Transthoracic echocardiography measures at the follow-up evaluation, according to the presence of dyspnea.

Measures	Overall Patients (*n* = 225)	No Dyspnea at FU (*n* = 110)	Dyspnea at FU (*n* = 115)	*p* Value
LV EDVi biplane (mL/m^2^) (median IQR (range))	51 (43, 58)	52 (46, 59)	50 (42, 57)	NS
LV EDVi 3D (mL/m^2^) (median IQR (range))	54 (47, 62)	56 (48, 62)	53 (46, 62)	NS
LV ESVi biplane (mL/m^2^) (median IQR (range))	20 (16, 23)	20 (16, 24)	20 (16, 22)	NS
LV ESVi 3D (mL/m^2^) (median IQR (range))	21 (18, 25)	21 (18, 25)	20 (18, 25)	NS
LV EF biplane (%) (median IQR (range))	61 (57, 65)	61 (57, 65)	61 (57, 64)	NS
LV EF 3D (%) (median IQR (range))	61 (58, 64)	61 (58, 63)	60 (57, 64)	NS
LV GLS (%) (median IQR (range))	−18.6 (−20.4, −17.0)	−18.6 (−20.8, −17.0)	−18.4 (−20.1, −26.7)	NS
LV E/A ratio (median IQR (range))	0.85 (0.71, 1.08)	0.85 (0.71, 1.08)	0.85 (0.70, 1.08)	NS
LV E/e’ ratio (median IQR (range))	7.5 (6.0, 9.1)	7.7 (6.0, 8.9)	7.1 (5.9, 9.7)	NS
Left atrial volume index (mL/m^2^) median IQR (range))	30 (25, 35)	30 (27, 36)	29 (24, 35)	NS
RV EDAi (cm^2^/m^2^) (median IQR (range))	11 (9, 12)	11 (10, 12)	11 (9, 12)	NS
RV ESAi (cm^2^/m^2^) (median IQR (range))	6 (5, 7)	6 (5, 7)	6 (5, 7)	NS
FAC (%) (median IQR (range))	44 (40, 47)	44 (40, 47)	44 (40, 48)	NS
RV EDVi 3D (mL/m^2^) (median IQR (range))	50 (41, 61)	53 (42, 61)	44 (39, 58)	NS
RV ESVi 3D (mL/m^2^) (median IQR (range))	23 (19, 30)	25 (20, 32)	23 (19, 29)	NS
RV EF 3D (%) (median IQR (range))	52 (47, 56)	52 (48, 56)	51 (47, 55)	NS
RV FWS (%) (median IQR (range))	−24.4(−27.0, −22.0)	−25.0 (−27.3, −22.0)	−24.0 (−26.0, −21.4)	NS
RV GLS (%) (median IQR (range))	−20.3 (−22.5, −18.4)	−20.4 (−22.6, −18.7)	−20.0 (−22.0, −18.2)	NS
RV GLS/sPAP (%/mmHg) (median IQR (range))	0.78 (0.97, 0.64)	0.78 (0.93, 0.66)	0.78 (1.04, 0.63)	NS
TAPSE (mm) (median IQR (range))	22 (20, 24)	22 (20, 24)	22 (19, 24)	NS
TAPSE/sPAP (mm/mmHg)	0.86 (0.71, 1.04)	0.82 (0.71, 1.01)	0.88 (0.71, 1.06)	NS
Probability of pulmonary hypertension (n (%))				NS
-Low	193 (93%)	96 (93%)	97 (92%)	
-Intermediate	11 (5.3%)	6 (5.8%)	5 (4.8%)	
-Intermediate-high	3 (1.4%)	1 (1%)	2 (1.9%)	
-High	1 (0.5%)	0 (0%)	1 (1%)	
sPAP (mmHg) (median IQR (range))	26 (21,29)	26 (22, 29)	25 (21, 29)	NS
PVR (WU) (median IQR (range))	1.73 (1.49, 1.99)	1.77 (1.5, 2.00)	1.71 (1.42, 1.98)	NS
AT (msec) (median IQR (range))	129 (116, 146)	130 (120, 145)	128 (114, 146)	NS

Acceleration time (AT); ejection fraction (EF); end-diastolic volume index (EDVi); end-systolic volume index (ESVi); fractional area change (FAC); interquartile range (IQR); left ventricle (LV); left ventricle global longitudinal strain (LVGLS); number (n); not significant (NS); pulmonary vascular resistance (PVR); right ventricle (RV); right ventricle end-diastolic area index (RVEDAi); right ventricle end-systolic area index (RVEDAi); right ventricle free-wall strain (RV FWS); right ventricle global longitudinal strain (RVGLS); systolic pulmonary artery pressure (sPAP); tricuspid annular plane excursion (TAPSE).

**Table 3:** Pulmonary functional test and 6MWT measures at the follow-up evaluation, according to the presence of dyspnea.

Measures	Total Patients (*n* = 225)	Dyspnea Absent at FU (*n* = 110)	Dyspnea Present at FU (*n* = 115)	*p* Value
VC (l) (median IQR (range))	3.56 (2.86, 4.18)	3.63 (3.05, 4.27)	3.34 (2.64, 4.05)	NS
VC (%) (median IQR (range))	102 (91, 114)	104 (93, 115)	101 (86, 113)	NS
TLC (l) (median IQR (range))	5.64 (4.64, 6.61)	5.85 (4.92, 6.66)	5.37 (4.41, 6.51)	0.044
TLC (%) (median IQR (range))	95 (86, 103)	96 (88, 103)	94 (83, 103)	NS
DLCO (mL/min/mmHg) (median IQR (range))	19 (14, 23)	20 (15, 24)	18 (14, 22)	0.036
DLCO (%) (median IQR (range))	76 (61, 87)	79 (66, 90)	73 (58, 84)	0.015
KCO (l) (median IQR (range))	3.63 (2.94, 4.12)	3.63 (2.99, 4.02)	3.63 (2.93, 4.16)	NS
MIP (cm H_2_O) (median IQR (range))	74 (56, 103)	83 (61, 103)	70 (53, 103)	NS
MEP (cm H_2_O) (median IQR (range))	90 (68, 113)	93 (73, 119)	85 (63, 104)	0.034
Tiffeneau index (%) (median IQR (range))	0.84 (0.79, 0.88)	0.84 (0.80, 0.88)	0.84 (0.79, 0.88)	NS
Distance at 6MWT (m) (median IQR (range))	420 (360, 480)	450 (385, 495)	420 (360, 480)	NS
Final SpO_2_ at 6MWT (%) (median IQR (range))	96 (95, 98)	97 (96, 98)	96 (94, 98)	NS
Absolute drop in SpO_2_ at 6MWT (%) (median IQR (range))	2 (1, 3)	2 (1, 3)	2 (1, 4)	NS

Carbon monoxide transfer coefficient (KCO); interquartile range (IQR); lung diffusion capacity for carbon monoxide (DLCO); maximum expiratory pressure (MEP); maximum inspiratory pressure (MIP); number (n); not significant (NS); peripheral capillary oxygen saturation (SpO_2_); six-minute walking test (6MWT); total lung capacity (TLC); vital capacity (VC).

**Table 4:** Acute predictors of persistent dyspnea.

	Univariable Analysis			Multivariable Analysis		
Variables	OR	95% CI	*p* Value	OR	95% CI	*p* Value
Age	1.00	0.98, 1.02	NS			
Gender (male)	0.58	0.33, 1.00	NS			
Smoke			NS			
-User	0.39	0.08, 1.43				
-Former smoker	1.45	0.84, 2.52				
BMI (kg/m^2^)	1.02	0.97, 1.07	NS	1.15	1.06, 1.28	0.001
Previous CVD	1.98	0.61, 7.61	NS			
Duration of hospitalization	1.02	1.00, 1.04	0.029	1.05	1.01, 1.10	0.005
Respiratory			NS			
Ventilation						
-NC-SM	0.54	0.19, 1.54				
-RM	0.50	0.08, 3.05				
-HFNC	0.32	0.13, 0.74				
-NIV	0.58	0.21, 1.59				
-MV	0.67	0.27, 1.61				
Troponin I	1.00	1.00, 1.00	NS			
CRP	1.00	1.00, 1.01	0.013	1.01	1.00, 1.02	0.025
D-dimer	1.00	1.00, 1.00	NS			
BNP	1.00	1.00, 1.00	NS			
Cardiovascular risk factors	0.99	0.56, 1.72	NS			

Body mass index (BMI); B-type natriuretic peptide (BNP); cardiovascular disease (CVD); CI (confidence interval); C-reactive protein (CRP); high flow nasal cannula (HFNC); mechanical ventilation (MV); nasal cannula (NC); non-invasive ventilation (NIV); non-rebreather mask (RM); not significant (NS); odds ratio (OR); simple face mask (SM).

**Table 5:** Association between persistent dyspnea and cardiopulmonary parameters at follow-up.

	Univariable Analysis			Multivariable Analysis		
Measures	OR	95% CI	*p* Value	OR	95% CI	*p* Value
Laboratory tests						
CRP	1.02	0.97, 1.07	NS			
D-dimer	1.00	1.00, 1.00	NS			
Troponin I	1.02	0.99, 1.07	NS			
BNP	1.00	1.00, 1.00	NS			
6MWT						
Distance	1.00	0.99, 1.00	0.038			
Final SpO_2_	0.88	0.78, 0.99	0.040			
Absolute drop in SpO_2_	1.12	0.97, 1.30	NS	1.37	1.13, 1.69	0.001
Pulmonary functional tests						
VC (l)	1.02	0.97, 1.13	NS			
VC (%)	0.99	0.97, 1.00	NS			
TLC (l)	1.01	0.96, 1.11	NS			
TLC (%)	0.98	0.96, 1.00	NS			
DLCO	0.98	0.94, 1.01	NS			
DLCO (%)	0.98	0.97, 1.00	0.007			
KCO (l)	1.06	0.95, 1.32	NS			
MIP	1.00	0.99, 1.00	NS			
MEP	0.99	0.98, 1.00	0.038			
Tiffeneau index	0.23	0.01, 8.41	NS			
Echocardiography						
LV EDVi biplane	0.98	0.96, 1.00	NS			
LV EDVi 3D	0.98	0.96, 1.01	NS			
LV ESVi biplane	0.97	0.93, 1.02	NS			
LV ESVi 3D	1.00	0.96, 1.04	NS			
LV EF biplane	0.99	0.94, 1.04	NS			
LV EF 3D	0.98	0.92, 1.04	NS			
LV GLS	1.06	0.97, 1.17	NS			
LV E/A ratio	1.76	0.86, 4.00	NS			
LV E/e’ ratio	1.05	0.94, 1.18	NS			
LAVi	0.98	0.95, 1.00	NS			
RV EDAi	0.91	0.79, 1.05	NS			
RV ESAi	0.88	0.72, 1.05	NS			
FAC	1.00	0.96, 1.05	NS			
RV EDVi 3D	0.98	0.96, 1.00	NS			
RV ESVi 3D	0.98	0.94, 1.02	NS			
RV EF 3D (%)	0.98	0.93, 1.02	NS			
RV FWS	1.03	0.98, 1.09	NS			
RV GLS	1.02	0.98, 1.08	NS	1.12	1.02, 1.25	0.016
RV GLS/sPAP	0.92	0.38, 2.18	NS	0.14	0.02, 0.86	0.034
TAPSE	0.96	0.90, 1.02	NS			
TAPSE/sPAP	1.23	0.45, 3.45	NS			
Probability of pulmonary hypertension			NS			
-Low	0.82	0.23, 2.83				
-Intermediate	1.98	0.19, 43.0				
-High	NA	NA				
sPAP	0.99	0.95, 1.03	NS			
PVR	0.83	0.46, 1.18	NS			
AT	0.83	0.46, 1.18	NS			

Acceleration time (AT); B-type natriuretic peptide (BNP); carbon monoxide transfer coefficient (KCO); confidence interval (CI); C-reactive protein (CRP); ejection fraction (EF); end-diastolic volume index (EDVi); end-systolic volume index (ESVi); fractional area change (FAC); left atrial volume index (LAVi); left ventricle (LV); left ventricle global longitudinal strain (LVGLS); lung diffusion capacity for carbon monoxide (DLCO); maximum expiratory pressure (MEP); maximum inspiratory pressure (MIP); not applicable (NA); not significant (NS); odds ratio (OD); peripheral capillary oxygen saturation (SpO_2_); pulmonary vascular resistance (PVR); right ventricle (RV); right ventricle end-diastolic area index (RVEDAi); right ventricle end-systolic area index (RVESAi); right ventricle free-wall strain (RV FWS); right ventricle global longitudinal strain (RVGLS); systolic pulmonary artery pressure (sPAP); six-minute walking test (6MWT); total lung capacity (TLC); tricuspid annular plane excursion (TAPSE); vital capacity (VC).

## Data Availability

The data presented in this study are available upon request from the corresponding author. The data are not publicly available due to privacy reasons.
